# Significance of 1,25-Dihydroxyvitamin D_3_ on Overall Mortality in Peritoneal Dialysis Patients with COVID-19

**DOI:** 10.3390/nu15092050

**Published:** 2023-04-24

**Authors:** Marko Baralić, Dragana Robajac, Ana Penezić, Voin Brković, Nikola Gligorijević, Ana Bontić, Jelena Pavlović, Jelena Nikolić, Goran Miljuš, Zorana Dobrijević, Miloš Šunderić, Lucia Pažitná, Jaroslav Katrlík, Olgica Nedić, Mirjana Laušević

**Affiliations:** 1 School of Medicine, University of Belgrade, 11000 Belgrade, Serbia; 2 Clinic of Nephrology, University Clinical Centre of Serbia, 11000 Belgrade, Serbia; 3 Institute for the Application of Nuclear Energy (INEP), University of Belgrade, 11080 Belgrade, Serbia; 4 Institute of Chemistry, Technology and Metallurgy National, Institute of the Republic of Serbia, University of Belgrade, 11000 Belgrade, Serbia; 5 Institute of Chemistry, Slovak Academy of Sciences, 84538 Bratislava, Slovakia

**Keywords:** calcitriol, COVID-19, peritoneal dialysis, glycosylation, mortality

## Abstract

In previous publications, we pointed out the importance of mannosylation of fibrinogen for the development of cardiovascular complications and fucosylation as a predictor of peritoneal membrane dysfunction in patients on peritoneal dialysis (PD). After a follow-up period of 30 months from the onset of the COVID-19 pandemic, we evaluated the significance of 1,25-dihydroxyvitamin D_3_ (calcitriol) therapy, primary disease, biochemical and hematologic analyzes, and previously performed glycan analysis by lectin-based microarray as predictors of mortality in this patient group. After univariate Cox regression analysis, diabetes mellitus (DM) and calcitriol therapy were found to be potential predictors of mortality. Additional multivariate Cox regression analysis confirmed that only DM was a predictor of mortality. Nevertheless, the use of calcitriol in therapy significantly reduced mortality in this patient group, as shown by the Kaplan–Meier survival curve. The presence of DM as a concomitant disease proved to be a strong predictor of fatal outcome in PD patients infected with SARS-CoV-2. This is the first study to indicate the importance and beneficial effect of calcitriol therapy on survival in PD patients with COVID-19 infection. In addition, this study points to the possibility that adverse thrombogenic events observed in PD patients during the pandemic may be caused by aberrant fibrinogen glycosylation.

## 1. Introduction

Peritoneal dialysis (PD), one of the methods of renal replacement therapy (RRT), is used by nearly 300,000 people worldwide [1]. The most common comorbidities in patients with end-stage renal disease (ESRD) are anemia in chronic disease and secondary hyperparathyroidism (SHPT), which is characterized by disturbances in calcium (Ca^2+^) and phosphate (PO_4_^−^) metabolism [2]. PD patients are treated with 1,25-dihydroxyvitamin D_3_ (calcitriol) when Ca^2+^ levels are above 2.65 mmol/L, PO_4_^−^ levels are below 0.80 mmol/L, and parathyroid hormone (PTH) levels are four times the upper reference value (above 260 ng/L). Because ESRD patients are at increased risk for severe SARS-CoV-2 infection, PD carries a lower epidemiologic risk because treatment is administered at home [3]. During the COVID-19 pandemic, a growing body of work pointed to the protective role of Vit D against severe forms of disease, inflammation, and prevention of death in the general population [4]. Adequate Vit D status has been shown to be a potential preventive factor in preventing the progression of COVID-19 pneumonia and inflammation due to its immunomodulatory role [5,6]. In addition to passive immunization, which has been performed in the last 2 years, an innate (adaptive) immune response plays an important role in preventing severe forms of the disease (e.g., acute respiratory distress syndrome, ARDS). Vit D has been shown to reduce the affinity of viral particles for binding to angiotensin-2 converting enzyme-2 (ACE2) on type I pneumocytes, thereby preventing the development of ARDS. In addition, Vit D decreases the production of pro-inflammatory cytokines and increases the production of anti-inflammatory cytokines and natural antimicrobial peptides, as well as the activation of non-specific immune cells, improving the clinical picture and disease progression [7].

Considering that COVID-19 is an inflammatory disease and that adverse thrombogenic events are the most common cause of death in patients with COVID-19 inflammation [8,9], we also investigated whether there is an association between aberrant fibrinogen glycosylation and thrombogenic events. Fibrinogen is an acute-phase reactant and a key player in the regulation of inflammation as well as the first factor in the coagulation cascade. This protein undergoes various post-translational modifications, the most important of which are oxidation and glycosylation [10]. Circulating fibrinogen concentrations are increased in COVID-19 patients, while blood clots are denser, stiffer, less porous, and exhibit altered polymerization and degradation properties, in part due to fibrinogen increased sialylation [11].

This study is a single center, prospective, observational study designed to decipher the potentially protective effect of calcitriol therapy on the survival of PD patients infected with COVID-19. Changes in fibrinogen glycosylation between PD patients with and without calcitriol therapy were also examined.

## 2. Materials and Methods

### 2.1. Study Group

This study is a prospective analysis of patients treated with peritoneal dialysis during the SARS-CoV-2 pandemic. All patients were regularly examined at the Clinic of Nephrology of the University Clinical Center of Serbia. The study was approved by the Ethics Committee of the University Clinical Center of Serbia and the University of Belgrade, Faculty of Medicine (No. 890/8). All respondents gave their written informed consent to participate in the study. The study itself was conducted in accordance with the Declaration of Helsinki and the Ethical Guidelines for Medical and Health Research, which include human trials. Only patients who did not have peritonitis or clinical and laboratory evidence of infection of the exit site (site of the peritoneal catheter) in the three months before sample collection were included in the study. Patients taking oral anticoagulants and/or antiplatelet agents were excluded from the study, as were patients with coagulopathy or hematologic malignancies. Patients with acute or chronic liver lesions were also excluded, i.e., all patients studied had negative virological status for hepatotropic viruses (anti-HCV and HbsAg), and in biochemical analyzes, aminotransferases (AST and ALT), gamma- GT, and bilirubin (direct and indirect) were within reference values. Only after the diagnosis of secondary hyperparathyroidism, patients were administered calcitriol. None of the patients had been vaccinated against SARS-CoV-2 at the beginning of the study or before the first episode of SARS-CoV-2 infection. During the pandemic, all patients performed PD treatment in their homes. After testing positive for SARS-CoV-2, patients were treated at the specialized COVID-19 hospital, where their health was monitored by nephrology specialists. Neither continuous veno-venous hemofiltration or continuous veno-venous hemodiafiltration were employed. If necessary, PD patients were transferred to hemodialysis. All patients with DM were on insulin therapy and not on oral antidiabetic drugs.

### 2.2. Samples

Data were obtained from the medical records, and all patients were treated with standard glucose solutions, whereas 6 patients also used glucose polymer (icodextrin) only for the longest dialysis shift. The values of biochemical parameters were measured at the control examination at the beginning of the pandemic. Serum levels of calcitriol were not monitored. Dialysis adequacy was expressed by urea clearance (Kt/V) and weekly creatinine clearance (weekly Ccr). The Baxter software package (Healthcare, Deerfield, IL, USA) was used for assessment.

### 2.3. Peritoneal Membrane Function

The Peritoneal Equilibration Test (PET) can be used to assess the transport properties of the peritoneal membrane. It determines the rate at which solutes are transported across the peritoneal membrane until equilibrium is established for a given substance on both sides of the peritoneal membrane, in the serum (circulation) and in the infused dialysis solution [12]. The test was performed according to the recommendations of Cnossen et al. [13].

### 2.4. Fibrinogen Isolation and Glycoanalysis

This was done according to the procedure published by Baralić at al [14]. Briefly, fibrinogen was isolated from 500 μL of plasma with ammonium sulfate solution at a final concentration of 20%. The precipitate was separated by centrifugation at 10,000× *g* for 5 min and dissolved in 50 mM phosphate buffer containing 150 mM sodium chloride (PBS, pH 7.4). Fibrinogen concentration was adjusted to 100 μg/mL with PBS. All samples were analyzed with a lectin-based protein microarray. Fibrinogen samples were printed on epoxysilane-coated microarray slides (NEXTERION Slide E, Schott, Germany) in eight identical subarrays using the piezoelectric printer sciFLEX-ARRAYER S1 equipped with the piezo dispensing capillary PDC 80 (Scienion AG, Berlin, Germany) at a temperature of 14 °C and humidity of 60%. After incubation at 4 °C for 2 h, the unoccupied reactive sites were blocked with 3% bovine serum albumin in PBS at 4 °C for 1 h. The excess of blocking agent was removed by washing, followed by incubation with biotinylated lectins (25 μg/mL in PBS containing 0.05% Tween 20-PBST) at 25 °C for 1 h (the list of lectins is given in [10]). After thorough washing with PBS, the bound lectins were allowed to interact with 0.5 μg/mL CF647-streptavidin conjugate in PBS at 25 °C for 15 min. The slides were then washed thoroughly with PBST and distilled water, dried (centrifugation), and scanned using the InnoScan^®^ 710 fluorescence scanner (Innopsys, Carbonne, France). The signals obtained were analyzed using Mapix^®^ 7.4.1 software (Innopsys, Carbonne, France).

### 2.5. Statistical Analysis

Methods of descriptive and inferential statistics were used. Of the methods of descriptive statistics, we have used measures of central tendency (arithmetic mean), measures of variability (standard deviation), and relative numbers. Continuous variables are presented as mean ± SD, and the normality of the distribution was tested with the Kolmogorov–Smirnov test. Univariate Cox proportional hazard analysis was used to identify predictors of fatal outcome during the follow-up period. Variables that showed significant prediction at a *p* value of less than 0.1 were included in the multivariate Cox model, using a stepwise forward method (likelihood ratio). The variables with a *p*-value of less than 0.05 were considered as independent predictors of mortality. Statistical analysis was performed using the SPSS software package, version 18.0 (SPSS Inc., Chicago, IL, USA).

## 3. Results

Fifty-two patients participated in the study, evenly divided between the sexes (26 women and 26 men). The basic biochemical findings and hematological characteristics are presented in the paper by Baralić et al. [10]. None of the patients included in this study were vaccinated at baseline, and during the follow-up period, all patients tested positive for SARS-CoV-2. The follow-up period had the median of 26 months with interquatile range (IQR) of 16 months (min/max 1–30).

At baseline, 21 patients (40%) were not on calcitirol therapy and were designated as group 1, while 31 patients (60%) were on calcitriol therapy and were designated as group 2. Calcitriol therapy in group 2 was introduced as regular therapy for the treatment of SHPT. Table 1 shows the stratification of patients in both groups according to gender, age, cause of ESRD and dialysis adequacy.

As shown in Table 1, the patients included in this study were evenly distributed according to sex, age, primary cause of ESRD, and duration of PD. All participants included in the study were nonsmokers. None of the parameters analyzed showed statistically significant differences between the study groups. We also compared these two patient groups with respect to their comorbidities before the onset of the pandemic (Table 2).

The results presented in Table 2 show that the prevalence of all comorbidities, except diabetes mellitus, was similar in both groups. Diabetes mellitus, however, was more prevalent in group 1 (without calcitriol therapy), representing a statistically significant difference (*p* = 0.023). In DM patients, levels of HbA1c were within a reference range: 5.95 ± 0.95 (4.6–8.7).

Within the defined groups, patients were compared based on their dialysis characteristics, and the results are shown in Table 3.

The analyzed parameters of dialysis did not differ between groups. In both study groups, patients were predominantly medium-fast glucose transporters and medium-slow creatinine transporters. The mean values for Kt/V and Ccr indicate PD as a competent method of RRT. There were also no differences between the groups with regard to the distribution of peritonitis episodes. Patients participating in the study were also compared on the basis of their biochemical parameters (Table 4).

As can be seen from the results presented, the groups studied had approximately equal values for most biochemical and hematological parameters, while there was a statistically significant difference only in the concentration of parathyroid hormone (*p* < 0.001). The higher PTH concentration observed in group 2 was an indicator for calcitriol administration.

Advanced cardiovascular events caused by thrombogenic events are the leading cause of death in PD patients. Therefore, we analyzed the differences in fibrinogen glycosylation between the two groups of patients involved in this study using samples collected before the onset of the pandemic. Fibrinogen glycosylation was analyzed using a lectin-based microarray, and the results are shown in Table 5.

A statistically significant difference between groups was found in the intensity of the signal originating from the interaction of lectin WGA with fibrinogen (*p* = 0.007). Taking into account the sugar specificity of WGA, it can be concluded that fibrinogen from patients in group 1 has a higher content of penultimate *N*-acetyl-D-glucosamine in the glycan component of this glycoprotein.

Of the total number of patients, 25 (89%) required inpatient COVID-19 treatment because of the need for oxygen therapy. Between March 2020 and September 2022, 18 patients (35%) died. All of them died in the acute phase of the disease and within 30 days of testing positive for SARS-CoV-2.

Analysis of PD patients COVID-19 survival.

**Figure 1:**
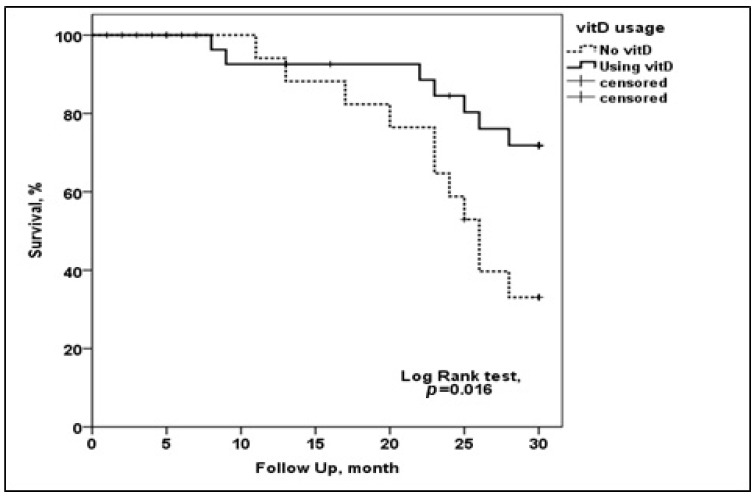
Kaplan–Meier curve of progression-free survival in PD patients with and without calcitriol therapy.

As shown in Figure 1, calcitriol administration had a positive effect on the survival of PD patients with COVID-19 infection. We also analyzed the number of lethal outcomes in the studied groups (Table 6).

The results (Table 6) also show that calcitriol therapy has a positive effect on the survival of PD patients with COVID-19 infection, as there is a statistically relevant difference in fatal outcome between the two groups (*p* = 0.027). To decipher which of the studied parameters could serve as a predictor of fatal outcome in PD patients with COVID-19, we performed a univariate Cox proportional hazard regression analysis (Table 7).

The obtained results suggest that the presence of DM and the administration of calcitriol could be considered relevant in predicting the fatal outcome of COVID-19 in PD patients. Additional multivariate Cox proportional hazard regression analysis for death prediction confirmed the presence of DM (as a comorbid condition) as a positive predictor of death from COVID-19 infection in PD patients (Table 8).

## 4. Discussion

Chronic kidney disease (CKD) is taking on the features of a pandemic, with a current prevalence of 843 million people worldwide [15]. This number is expected to increase as more patients are diagnosed with diabetes mellitus (DM) and hypertension (HTN), the main causes of ESRD [16]. ESRD patients are 10- to 30-fold more likely to develop COVID-19 pneumonia than the general population [17]. Therefore, it is important to elucidate the mechanisms and conditions under which ESRD patients develop a severe form (i.e., ARDS) of the disease. The significantly high incidence of ARDS is the result of the influence of stress, chronic inflammation, and various metabolic disorders in ESRD patients. In addition, hypovitaminosis D, metabolic disorders Ca^2+^ and PO_4_^−^, hyperglycemia, dyslipidemia, and others contribute to a severe form of COVID-19 [18]. Compared with HD, PD is a much less common model of RRT. While patients who travel to HD several times a week and ride in ambulances with other patients are at higher risk of infection, PD is performed at home and is therefore the model of choice for ESRD patients during the pandemic [19,20]. It is difficult to decide which of the RRT methods is better, because the treatment should be tailored to the individual patient. Fifty-two patients, evenly distributed by gender and age, participated in our study. During the follow-up period of 30 months after the pandemic outbreak, all of them tested positive for COVID-19. Nearly 45% (25) required inpatient COVID-19 treatment due to the need for oxygen therapy. Between March 2020 and September 2022, 18 patients (35%) died—all in the acute phase of the disease. This is consistent with data in the literature [8,9,21]. They were treated in the ICU because they required mechanical ventilation, and the main causes of death were adverse thrombogenic events and inflammation.

The presence of HTN and DM, either as primary or associated diseases, was found to be the most important predictive factor for the development of severe forms of SARS-CoV-2 infection [22]. The results are consistent with most of the studies presented so far showing the influence of HTN, DM and obesity as the most important risk factors for the exacerbation of COVID-19 infection [23]. These factors are also the most important predictors for the development of adverse cardiovascular complications and fatal outcomes. Diabetes mellitus significantly contributed to the unfavorable outcome of PD patients treated for COVID-19 pneumonia. This is consistent with data published for HD patients [24,25].

SHPT with impaired Ca^2+^ and PO_4_^+^ metabolism is a common complication in patients with ESRD, which may occur even in earlier stages of CKD. The disorder develops as a result of inadequate activation of Vit D at the level of proximal tubule cells and subsequent hypocalcemia and hyperphosphatemia, which stimulate the secretion of PTH [5,26]. Our study groups differed significantly in PTH levels. This was expected because one of the groups consisted of patients with SHPT who initially received calcitriol therapy for the reasons mentioned above. Hypovitaminosis D leads to susceptibility to obesity and insulin resistance, one of the most important factors in the development of adverse cardiovascular complications [27,28]. Hypervitaminosis D, on the other hand, inhibits the proliferation of B and T lymphocytes and reduces the production of proinflammatory cytokines, resulting in lower levels of inflammatory markers in patients treated with Vit D supplements [29]. Vit D is necessary for normal bone development and growth. It has a protective effect against the action of free oxygen species and anti-inflammatory cytokines, preventing the formation of a cytokine storm, the initiation of the coagulation cascade, and the conditioning of thrombogenic events, which have been shown to lead to severe COVID-19 infection, multi-organ distress syndrome, and death [30]. Almost half of COVID-19 patients with hypovitaminosis D before infection developed a severe form of the disease [31]. There are reports of the protective effect of Vit D in preventing acute viral respiratory infections [32,33], but its contribution to reducing the risk of severe forms of COVID-19 and fatal outcome is controversial [34].

The examination of fibrinogen glycosylation showed a statistically significant difference for WGA lectin signal, suggesting that important structural parts of this protein differ significantly between our study groups. The increase in fibrinogen sialylation in COVID-19 patients is associated with the formation of a denser, less porous, and more thrombogenic clots [11]. It is plausible that the increase in *N*-acetylglucosamine content observed in our study, in addition to the higher fibrinogen concentration, contributes to the observed clot characteristics. We found no significant difference between the studied groups in Ca^2+^ and PO_4_^−^ levels, confirming that hyperphosphatemia is not a good predictor of unfavorable outcomes in patients on PD [35,36]. However, we found that the presence of DM as a comorbidity was a predictor of fatal outcome in these patients.

Therapeutic calcitriol administration (treatment of SHPT) had a positive effect on patient survival, as the number of patients who died was twofold lower compared to those who did not receive calcitriol. The importance of Vit D in the survival of COVID-19 infections has already been reported in the general population and in hemodialysis patients [21,33,37,38]. This is the first report showing that this effect also occurs in patients treated with PD.

Previous studies have shown that platelet count is important for the development of thrombotic complications in patients with SARS-CoV-2 infection [39]. In this study, we compared PD patients divided into two groups based on the inclusion of calcitriol in therapy and found no significant difference between the studied groups in terms of platelet count. Dialysis adequacy parameters (Kt/V and Ccr) were also not significantly different between the groups, suggesting the suitability of the RRT method [40]. Patient age and hypoalbuminemia are significant predictors of unfavorable outcome in patients on dialysis treatment [41]. Nevertheless, these parameters did not differ between our groups, due to study design, and can be ruled out as a cause of the observed differences between groups in terms of survival. Finally, we must point to some study limitations, i.e., limited number of participants, small subgroups, missing information on serum levels of vitamin D.

## 5. Conclusions

This is the first study to confirm that the presence of DM as a comorbidity is a strong predictor of death in PD patients with COVID-19. Univariate Cox regression analysis excluded known negative predictors of mortality such as age, serum albumin and phosphate concentration. At the same time, this study showed the calcitriol intake for survival of patients on PD with SARS-CoV-2 infection. Moreover, this study indicated the possibility that the unfavorable outcomes observed in PD patients during the pandemic might somehow be related to aberrant fibrinogen glycosylation. More detailed research investigating fibrinogen glycosylation in other groups of patients prone to thrombosis, and in SARS-CoV-2-positive patients only, should provide us with a more comprehensive answer.

## Figures and Tables

**Table 1:** Grouping of patients according to the primary disease, age and length of dialysis, group 1 (patients without calcitriol therapy) and group 2 (patients on calcitriol therapy). *p*-statistical significance.

		Group 1 (*n* = 21)	Group 2 (*n* = 31)	*p*-Value
Male, *n* (%)		11 (52.4%)	15 (48.4%)	0.777
BMI, mean ± SD		23.8 ± 1.17	23.6 ± 4.80	0.877
Cause of ESRD	1	9 (42.9%)	7 (22.6%)	0.352
	2	3 (14.3%)	10 (32.2%)	
	3	6 (28.5%)	5 (16.2%)	
	4	1 (4.8%)	3 (9.7%)	
	5	2 (9.5%)	4 (12.9%)	
	6	0 (0%)	2 (6.4%)	
Average duration of PD (months)		35	26	0.526
Age ± SD		59 ± 14.5	65 ± 12.6	0.107

1—nephroangiosclerosis; 2—diabetic nephropathy, 3—glomerulopathies, 4—autosomal dominant polycystic kidney disease, 5—tubulointerstitial nephritis, 6—obstructive uropathy.

**Table 2:** Prevalence of the most common associated diseases in patients treated with PD divided into two groups according to the use of calcitriol. Group 1 (patients without calcitriol therapy); Group 2 (patients on therapy with calcitriol); *p*-statistical significance.

Associated Diseases	Group 1 (*n* = 21)	Group 2 (*n* = 31)	*p*-Value
CVS, *n* (%)	4 (19%)	2 (5.6%)	0.170
DM, *n* (%)	12 (57.1%)	8 (25.8%)	0.023 *
HTN, *n* (%)	20 (95.2%)	29 (93.5%)	0.798
CMP, *n* (%)	12 (57.1%)	11 (35.5%)	0.123
AIM, *n* (%)	5 (23.8%)	7 (22.6%)	0.198

CVS—cerebrovascular stroke, DM—diabetes mellitus, HTN—hypertension, CMP—cardiomyopathy, AIM—acute myocardial infarction. Statistically significant difference (*p*-value < 0.05) is given in bold and marked with an asterisk.

**Table 3:** Dialysis characteristics of patients: group 1 (patients without calcitriol therapy); Group 2 (patients on calcitriol therapy); *p*-statistical significance.

Dialysis Characteristics	Group 1 (*n* = 21)	Group 2 (*n* = 31)	*p*-Value
Number of peritonitis, *n* (%)			
0	11 (52.4%)	21 (67.7%)	0.305
1	8 (38.1%)	6 (19.4%)	
2	2 (9.5%)	2 (6.5%)	
4	0 (0%)	2 (6.5%)	
Kt/V, mean ± SD	2.29 ± 0.57	2.38 ± 0.51	0.561
Ccr L/w, mean ± SD	74 ± 17.7	82 ± 20.7	0.169
PET-gly, mean ± SD	0.44 ± 0,99	0.47 ± 0.104	0.237
PET-Cr, mean ± SD	0.66 ± 0.075	0.61 ± 0.124	0.109

Kt/V—urea clearance; Ccr—total weekly creatinine clearance; PET-gly—peritoneal equilibration test with glucose; PET-Cr—peritoneal equilibration test with creatinine.

**Table 4:** Biochemical and hematological characteristics of patients at the beginning of the pandemic. Group 1—patients without calcitriol therapy; Group 2—patients on calcitriol therapy; *p*-statistical significance.

Biochemical and Hematological Characteristics	Group 1 (*n* = 21)	Group 2 (*n* = 31)	*p*-Value
PTH ng/L, median (IQR)	187 (265)	615 (636)	<0.001 *
Hb, mean ± SD	102 ± 14.45	105 ± 17.39	0.518
Plt, mean ± SD	271 ± 91.99	233 ± 70.96	0.099
Gly mmol/L, mean ± SD	7.6 ± 5.73	6.1 ± 1.91	0.176
HbA1c %, mean ± SD	5.8 ± 0.88	5.4 ± 0.55	0.069
Ur mmol/L, mean ± SD	16.2 ± 5.95	17.1 ± 4.65	0.537
Cr mmol/L, mean ± SD	667 ± 147	632 ± 164.7	0.434
Alb g/L, mean ± SD	36 ± 3.99	38 ± 4.64	0.108
Fng g/L, mean ± SD	4.7 ± 0.95	4.6 ± 0.99	0.671
Ferritin μmol/L, median (IQR)	314 (295)	218 (278)	0.526

PTH—parathormone, Hb—hemoglobin, Plt—platelets, gly—glucose, Ur—urea, Cr—creatinine, Alb—albumin, Fng—fibrinogen. Statistically significant difference (*p*-value < 0.05) is given in bold and marked with an asterisk.

**Table 5:** Analysis of fibrinogen glycosylation performed using lectin-based glycoprotein microarray. Group 1—patients without calcitriol therapy, Group 2—patients on calcitriol therapy, *p*-statistical significance. All values are given as mean ± SD.

Lectin	Group 1 (*n* = 21)	Group 2 (*n* = 31)	*p*-Value
SNA	1961.4 ± 444.89	1959.6 ± 368.14	0.988
PHA-L	25.7 ± 14.96	20.0 ± 4.70	0.060
WGA	86.2 ± 29.71	67.9 ± 15.3	0.007 *
AAL	667.0 ± 173.80	594.0 ± 148.9	0.117
PhoSL	627.4 ± 92.4	600.7 ± 67.50	0.243
GSL	48.1 ± 20.74	39.6 ± 15.14	0.098
GNL	30.8 ± 7.67	26.9 ± 8.84	0.110

SNA—Sambucus nigra agglutinin, PHA-L—Phaseolus vulgaris agglutinin, WGA—Wheat germ agglutinin, AAL—Aleuria aurantia lectin, PhoSL—Pholiota squarrosa lectin, GSL—Griffonia simplicifolia lectin, GNL—Galanthus nivalis lectin. Statistically significant difference (*p*-value < 0.05) is given in bold and marked with an asterisk.

**Table 6:** Comparison of lethal outcome in patients between study groups. Group 1—patients without calcitriol therapy, Group 2—patients on calcitriol therapy. Statistically significant difference (*p*-value less than 0.05) is given in bold and marked with an asterisk.

	Group 1 (*n* = 21)	Group 2 (*n* = 31)	*p*-Value
Died, *n* (%)	11 (52.4%)	7 (22.6%)	0.027 *
DM, died	6	6	0.316
	**Survivors (*n* = 34)**	**Non-Survivors (*n* = 18)**	***p*** **-Value**
Calcitriol usage, *n* (%)	24 (70.6%)	7 (38.9%)	0.027 *
Presence of DM, *n* (%)	8 (23.5%)	12 (66.7%)	0.02 *

**Table 7:** Univariate Cox proportional hazards regression analysis for death prediction within studied population. *p*-statistical significance.

	B	*p*-Value	Hazard Ratio	95% CI (Lower–Upper)
Male	0.178	0.707	1.195	0.471–3.030
Age	−0.013	0.416	0.987	0.957–1.018
Duration on PD	0.008	0.196	1.008	0.996–1.020
DM	1.443	0.004 *	4.235	1.574–11.397
PTH concentration	0.001	0.679	1.000	0.999–1.001
Interaction with WGA	−0.002	0.809	0.998	0.978–1.018
Calcitriol usage	−1.102	0.023 *	0.332	0.128–0.862
Kt/V	−0.523	0.285	0.593	0.227–1.546
Alb	−0.098	0.119	0.906	0.801–1.026
PO_4_^−^	0.041	0.956	1.041	0.247–4.392
Ca^2+^	−0.637	0.520	0.529	0.076–3.690
CVS	0.220	0.770	1.246	0.285–5.446
HTN	0.411	0.690	1.508	0.200–11.337
CMP	0.377	0.427	1.458	0.576–3.692
AIM	0.565	0.285	1.759	0.624–4.955

CVS—cerebrovascular stroke, HTN—hypertension, CMP—cardiomyopathy, AIM—acute myocardial infarction. Statistically significant difference (*p*-value < 0.05) is given in bold and marked with an asterisk.

**Table 8:** Multivariate Cox proportional hazards regression analysis for death prediction among study population. *p*-statistical significance.

	B	*p*-Value	Hazard Ratio	95% CI (Lower–Upper)
DM	1.443	0.004 *	4.235	1.574–11.397
Calcitriol usage	-	0.188	-	-

Statistically significant difference (*p*-value < 0.05) is given in bold and marked with an asterisk.

## Data Availability

Not applicable.
